# Elevated serum lipoprotein(a) is significantly associated with angiographic progression of coronary artery disease

**DOI:** 10.1002/clc.23718

**Published:** 2021-08-25

**Authors:** Xing Shui, Zheqi Wen, Zefeng Chen, Xujing Xie, Yongxia Wu, Binghan Zheng, Zhen Wu, Lin Chen

**Affiliations:** ^1^ Department of Cardiovascular Medicine, The Third Affiliated Hospital Sun Yat‐sen University Guangzhou China; ^2^ Department of Cardiac Care Unit, The Third Affiliated Hospital Sun Yat‐sen University Guangzhou China

**Keywords:** body mass index, coronary artery disease, Gensini score, lipoprotein(a), progression

## Abstract

**Background:**

Lipoprotein(a)[Lp(a)] has been considered as an independent risk factor for coronary artery disease (CAD). The present study aimed to evaluate the association between baseline serum Lp(a) and CAD progression determined by angiographic score.

**Methods:**

A total of 814 patients who had undergone two or more coronary computed tomography angiography at least 6 months apart were consecutively enrolled and the coronary severity was determined by the Gensini score system. Patients were stratified into two groups according to Lp(a)>300 mg/L and Lp(a) ≤ 300 mg/L or classified as “progressors” and “non‐progressors” based on the Gensini score rate of change per year. The association of continuous Lp(a) and Lp(a)>300 mg/L with CAD progression were respectively assessed by logistic regression analysis. Moreover, further evaluation of those association was performed in subgroups of the study population.

**Results:**

Patients in the “progressors” group had significant higher Lp(a) levels. Furthermore, the multivariate logistic regression analysis showed that elevated Lp(a) (odds ratio [OR]: 1.451, 95% confidence interval [CI]: 1.177–1.789, *p*<.001) and Lp(a)>300 mg/L (OR:1.642, 95% CI:1.018–2.649, *p* = .042) were positively associated with CAD progression after adjusting for confounding factors. In addition, those relation seemed to be more prominent in subjects with lower body mass index (OR: 1.880, 95% CI: 1.224–2.888, *p* for interaction = .060).

**Conclusions:**

Elevated baseline serum Lp(a) is positively and independently associated with angiographic progression of CAD, particularly in participants with relatively low body mass index. Therefore, Lp(a) could be a potent risk factor for CAD progression, assisting in early risk stratification in cardiovascular patients.

## 1 INTRODUCTION

Coronary artery disease (CAD) is still a major cause of death worldwide. Despite great advances in its diagnosis, treatment, and prevention, adverse cardiovascular events do not seem to decline on the background of optimal medical therapy.^1^ The importance of identifying residual risk and screening the coronary atherosclerotic progression for early prevention remains undisputed.

Lipoprotein(a)[Lp(a)] is composed of a low‐density lipoprotein(LDL)‐like particle with its apolipoprotein (apo)B100, which covalently bound to a characteristic glycoprotein apolipoprotein a[apo(a)] by a disulfide bond.^2^ Because of its proatherogenic, proinflammatory and prothrombotic properties, previous studies demonstrated that Lp(a) involved in the pathophysiological process of coronary atherosclerosis.^3^, ^4^, ^5^ Meanwhile large amounts of evidence from epidemiological studies,^6^ mendelian randomization analysis,^7^ and genome‐wide association studies^8^ identified that Lp(a) was an independent risk factor for primary and secondary prevention of CAD. However, the correlation between Lp(a) and coronary atherosclerosis progression remains controversial. Previously, two study groups assessed the predictive utility of Lp(a) in coronary atherosclerosis progression by intravascular ultrasound, opposite results were obtained.^9^, ^10^ With the development of computed tomography technology, coronary computed tomography angiography (CTA) has been widely used for non‐invasive assessment of coronary atherosclerosis at outpatient department. Compared with invasive coronary angiography, the predictive value of coronary artery stenosis by coronary CTA had high sensitivity and specificity.^11^ Moreover, coronary CTA was more available and convenient for clinical application than invasive intravascular ultrasound.

Hence, in the present study, we aimed to analyze the association between baseline Lp(a) levels and angiographic progression of CAD by Gensini score system according to the results of coronary CTA in suspected CAD patients at outpatient department.

## 2 METHODS

### 2.1 Study population

**FIGURE 1:**
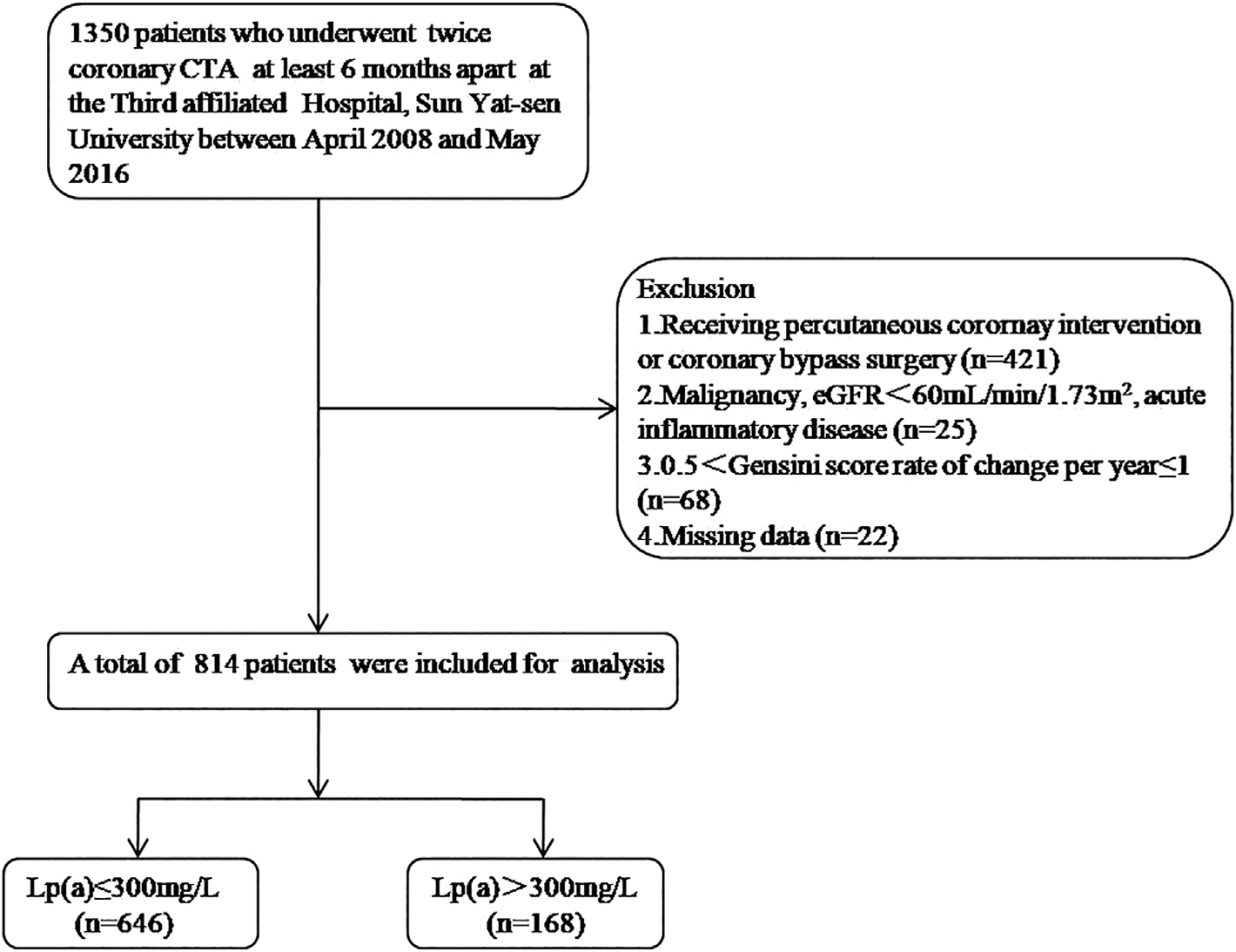
Flow chart of the current study. CTA, computed tomography angiography; eGFR, estimated glomerular filtration rate

This was a retrospective longitudinal study conducted at the Third Affiliated hospital, Sun Yat‐sen University between April 2008 and May 2016. The study subjects comprised of 1350 cardiovascular patients who underwent twice‐coronary CTA at least 6 months apart at outpatient department were enrolled. Patients initially received coronary CTA examination due to angina‐like symptoms, an abnormal ECG (including abnormal Q wave, ST‐T changes, or new complete left bundle branch block), an abnormal echocardiogram, positive treadmill exercise test or elevated cardiovascular risk. Based on the severity of angina‐like symptoms or coronary CTA images, some of them were admitted for further treatment. Among them, 421 patients receiving percutaneous coronary intervention or coronary bypass surgery were excluded. In addition, other exclusion criteria included patients with malignancy, estimated glomerular filtration rate(eGFR)<60 ml/min/1.73m^2^, acute inflammatory disease, 0.5<Gensini score rate of change per year≤1, or missing data. Eventually, 814 patients were analyzed. (Figure 1) This study was in line with the medical ethics standards of the 1975 Declaration of Helsinki and approved by the Ethics Committee of The Third Affiliated Hospital, Sun Yat‐sen University. Written informed consent was obtained from each patient.

### 2.2 Clinical and laboratory parameters

Clinical parameters including age, sex, height, weight, systolic and diastolic blood pressure (DBP), heart rate, comorbidities, and medications were collected at outpatient or admission. Body mass index (BMI) was calculated from weight in kg divided by height in m^2^. Hypertension (HTN) was defined as systolic blood pressure (SBP) ≥ 140 mm Hg and/or diastolic blood pressure ≥90 mmHg or currently using antihypertensive agents. Diabetes (DM) was defined as fasting serum glucose≥7.0 mmol/L or random serum glucose≥11.1 mmol/L or the 2‐h serum glucose of the oral glucose tolerance test≥11.1 mmol/L or using anti‐diabetic medication. Smoking status was defined as a person who smoked at the time of initial coronary CTA or who had quit smoking within the year before initial coronary CTA. Obstructive CAD was defined as coronary stenosis of 50% or more in the epicardial coronary artery determined by initial coronary CTA.

Blood samples were obtained from each patient in the fasting state at outpatient or after admission and then tested in laboratory medicine as soon as possible. The serum lipid profiles including total cholesterol (TC), triglycerides (TG), low density lipoprotein cholesterol (LDL‐C), high density lipoprotein cholesterol (HDL‐C), apoA1, apoB100, and Lp(a) were determined by automatic biochemical analyzer (Hitachi 7600, Tokyo, Japan). In addition, in detail, TC and TG were measured with enzyme colorimetry method, while LDL‐C and HDL‐C were determined by the direct method. ApoA1 and apoB100 were measured with immunoturbidimetry method. The serum concentration of Lp(a) was analyzed using latex immunoturbidimetry method. Due to racial differences, the optimal risk cutoffs for cardiovascular disease were still not clearly determined. The European Atherosclerosis Society proposed Lp(a) <50 mg/dl as the appropriate cutoffs, while China and the United States recommended Lp(a) <30 mg/dl as a desirable level.^12^ According to Guidelines for the Prevention and Treatment of Dyslipidemia in Chinese Adult,^13^ 300 mg/L was selected as a cutoff value in our center. The normal range was 0‐300 mg/L. Non‐HDL‐C levels were calculated as TC minus HDL‐C levels.

### 2.3 Coronary computed tomography angiography and Gensini score assessment

The 320‐slice coronary CTA was performed by a predefined standard operating procedure as previously described.^14^ Before coronary CTA examination, oral metoprolol tablets (50–100mm Hg, Astrazeneca)were given to patients with a resting heart rate>70 bpm. All patients were in the supine position and held their breath during the imaging process. Isovue‐370 (50–100 ml, Bracco Diagnostics, Guangzhou, China) was injected intravenously at a flow rate of 6.0 ml/s, followed by a 20 ml saline flush at a flow rate of 4.0 ml/s. 320‐slice coronary CTA was performed with 0.5‐mm detector element, 350 ms of gantry rotation time, and up to 16 cm of coverage in Z direction. Tube voltages were set at 100–135 kV and the maximal tube currents were set at 400–580 mA.

All coronary CTA images were reconstructed and analyzed using a dedicated workstation (VitreafX Version 2.1, TOSHIBA) by experienced radiologists, which in accordance with the Society of Cardiovascular Computed Tomography guidelines.^15^ The severity of coronary artery was assessed by angiographic scoring system: the Gensini score system, which were calculated by three experienced interventional cardiologists. Based on the degree of luminal narrowing, severity scores indicated angiographic stenosis of coronary artery segment were 1, 2, 4, 8, 16, and 32 for 0%–25%, 26%–50%, 51%–75%, 76%–90%, 91%–99%, and 100%, respectively.^16^ Given the variation in time during the coronary CTAs, the Gensini score rate of change per year was used to define angiographic CAD progression as detailed elsewhere.^17^ In brief, subjects were arbitrarily categorized as “progressors” and “non‐progressors” based on a Gensini score rate of change of >1 or ≤ 0.5 points/year, respectively. Subject with a Gensini score rate of change between 0.5< and ≤ 1 points/year were excluded as “gray area” in the present study.

### 2.4 Statistical analysis

All statistical analyses were performed using SPSS 25.0 software (IBM Corporation, Armonk, NY) for Windows. Continuous variables were shown as mean ± *SD*, or median with interquartile range, as appropriate. Student *t* test or Mann–Whitney U‐test was used to determine the differences in continuous variables between groups. Categorical variables were expressed as frequencies with percentage and analyzed by Chi square tests. Logistic regression analyses were conducted to explore the association between baseline Lp(a) and angiographic progression of CAD. Firstly, univariate logistic regression analyses were performed. Baseline Lp(a) was analyzed as a continuous variable or a categorical variable with a cutoff value as 300 mg/L according to Guidelines for the Prevention and Treatment of Dyslipidemia in Chinese Adult,^13^ respectively. Since the distribution of baseline Lp(a) showed positive skewing, it was logarithmically transformed when analyzed as a continuous variable. Therefore, the results were showed by odds ratio(OR) per log‐unit increase. Secondly, Multivariate logistic regression analyses were performed in three different models to adjust potential confounders. Covariates selection of multivariate logistic regression models was done by stepwise forward method and threshold values for F‐to‐enter and F‐to‐remove were 0.05 and 0.1, respectively. Model 1 was adjusted for age and gender. In Model 2, it was additionally adjusted for BMI, smoking status, history of HTN, DM, and initially obstructive CAD. In Model 3, we added HbA1c, fasting plasma glucose(FPG), HDL‐C, non‐HDL, LDL‐C, creatinine, uric acid (UA), and white cell counts (WBC) as confounding factors to assess the independent relation between Lp(a) and CAD progression. Thirdly, subgroup analyses were conducted after categorizing the patients according to sex, age, BMI, smoking status, history of HTN, DM, and initially obstructive CAD. In subgroup analyses, OR was obtained by log‐transformed continuous variable of Lp(a) after adjustment of covariates in Model 3. *p*‐value of <.05 was defined as statistically significant.

## 3 RESULTS

### 3.1 Baseline characteristics of subjects

A total of 814 eligible patients were entered into this study. The baseline serum Lp(a) values ranged from 3.00 to 1575.00 mg/L (median = 133.65 mg/L, interquartile range = 68.10–254.75 mg/L). Table 1 shows the baseline clinical characteristics of the study subjects. Patients were stratified into two groups according to Lp(a)>300 mg/L and Lp(a) ≤ 300 mg/L. Several baseline clinical and biochemical characteristics were compared between groups. Serum TC, HDL‐C, Non‐HDL‐C, and LDL‐C levels increased in the higher Lp(a) group. However, no significant difference was observed in demographic characteristics, clinical comorbidities, medications, as well as serum levels of TG, ApoA1, ApoB100, FPG, HbA1c, creatinine, eGFR, UA, and WBC.

**TABLE 1:** Baseline characteristics according to serum Lp(a) levels

Variables	Lp(a) ≤300 mg/L(*n* = 646)	Lp(a) >300 mg/L(*n* = 168)	*p*
Age, year	63.02 ± 10.82	63.99 ± 10.39	.297
Male, *n*(%)	340 (52.6)	84 (50.0)	.543
BMI, kg/m^2^	24.61 ± 3.10	24.11 ± 2.81	.058
Smoking, *n*(%)	175 (30.5)	38 (26.6)	.359
Hypertension, *n*(%)	426 (66.3)	112 (66.7)	.919
Diabetes, *n*(%)	261 (40.5)	62 (36.9)	.401
O‐CAD, *n*(%)	226 (35.0)	51 (30.4)	.259
SBP, mm Hg	140.72 ± 21.70	141.62 ± 19.23	.700
DBP, mm Hg	78.55 ± 12.07	76.64 ± 10.90	.164
HR, bpm	76.21 ± 12.21	77.95 ± 11.09	.187
**Laboratory tests**			
TC, mmol/L	4.87 ± 1.16	5.18 ± 1.19	.002^a^
TG, mmol/L	1.96 ± 1.82	1.75 ± 1.09	.175
HDL‐C, mmol/L	1.16 ± 0.30	1.23 ± 0.35	.004^a^
LDL‐C, mmol/L	3.00 ± 0.94	3.32 ± 1.01	<.001^a^
Non‐HDL, mmol/L	3.72 ± 1.11	3.95 ± 1.09	.016^a^
ApoA1, g/L	1.35 ± 0.27	1.36 ± 0.26	.692
ApoB100, g/L	1.04 ± 0.37	1.10 ± 0.36	.088
FPG, mmol/L	6.24 ± 3.01	6.36 ± 2.67	.657
HbA1c, (%)	6.63 ± 1.64	6.62 ± 1.70	.955
Creatinine, umol/L	76.15 ± 20.20	75.36 ± 19.55	.651
eGFR, ml/min/1.73m^2^	85.11 ± 47.58	83.89 ± 44.64	.765
UA, umol/L	371.86 ± 111.41	367.62 ± 111.94	.662
WBC, ×10E9/L	6.87 ± 1.74	6.85 ± 1.65	.906
**Medication**			
Anti‐platelet drug, *n*(%)	545 (84.4)	150 (89.3)	.108
Statins, *n*(%)	548 (84.8)	144 (85.7)	.775
ACEI/ARB, *n*(%)	418 (64.7)	116 (69.0)	.291
β‐blocker, *n*(%)	354 (54.8)	100 (59.5)	.272
OAD, *n*(%)	218 (33.7)	51 (30.4)	.405
Insulin, *n*(%)	96 (14.9)	28 (16.7)	.562

Abbreviations: AECI, angiotensin converting enzyme inhibitors; ARB, angiotensin receptor blocker; BMI, body mass index; DBP, diastolic blood pressure; eGFR, estimated glomerular filtration rate; FPG, fasting plasma glucose; HDL‐C, high‐density lipoprotein cholesterol; HR, heart rate; LDL‐C, low‐density lipoprotein cholesterol; OAD, oral anti‐diabetic drugs; O‐CAD, initially obstructive coronary artery disease; SBP, systolic blood pressure; TC, total cholesterol; TG, triglycerides; UA, uric acid; WBC, white cell counts.

^a^ *p*<.05.

### 3.2 Impact of clinical variables on coronary artery disease progression

Subjects were divided into two groups on the basis of the presence of angiographic CAD progression(a Gensini rate of change of >1 or ≤ 0.5 points/year was considered as “progressors” or “non‐progressors,” respectively). As is shown in Table S1, patients in “progressors” group were older; more often men and smoking; more likely to have history of HTN and initially obstructive CAD; and more likely to have elevated baseline serum Lp(a), ApoB100, HbA1c and decreased HDL‐C and ApoA1. In addition, the incidence of Lp(a)>300 mg/L was significantly higher in progressors group (25.6% vs. 18.3%, *p* = .018).

### 3.3 Gensini score at baseline and follow‐up according to baseline serum Lp(a) levels

Table 2 summarizes baseline and follow‐up changes in Gensini score. The initial Gensini score of all subjects was 7.23 ± 11.62 based on a per‐patients analysis. The absolute change of Gensini score was 2.31 ± 9.44 after a median follow‐up period of 2.42 (IQR: 1.48–3.68) year. When the Gensini score rate of change per year was used to define CAD progression, there were 258(31.7%) patients with CAD progression (a Gensini rate of change of >1 points/year), while the other 556 (68.3%) patients did not have significant progression (a Gensini rate of change of ≤0.5 points/year). Moreover, the incidence of CAD progression was statistically higher in patients with Lp(a)>300 mg/L(39.3% vs. 29.7%, *p* = .018).

**TABLE 2:** Baseline and follow‐up Gensini score related parameters according to serum Lp(a) levels

Variables	Total (*n* = 814)	Lp(a) ≤ 300 mg/L (*n* = 646)	Lp(a)>300 mg/L (*n* = 168)	*p*
Initial Gensini score	7.23 ± 11.62	7.25 ± 11.92	7.13 ± 10.38	.908
Changes in Gensini score	2.31 ± 9.44	1.95 ± 8.97	3.69 ± 11.00	.033^a^
Observation time, year	2.42 (1.48,3.68)	2.40 (1.50,3.65)	2.53 (1.30,3.89)	.891
Progression, n(%)	258 (31.7)	192 (29.7)	66 (39.3)	.018^a^

^a^ *p*<0.05.

### 3.4 Association of baseline Lp(a) levels with coronary artery disease progression

As a log‐transformed continuous variable, univariate logistic regression analysis demonstrated that increasing Lp(a) level was positively associated with CAD progression (OR 1.287, 95% CI 1.101–1.503 *p* = .002). In the multivariate analysis adjusting for age and sex in Model 1 showed the odds ratio for progression was 1.284(95% CI 1.095–1.506 *p* = .002). In Model 2, results seemed to enhanced these correlation after adjustment of clinical comorbidities, smoking status and BMI (OR 1.330, 95% CI 1.120–1.580; *p* = .001). In Model 3 including other serum lipids, HbA1c, FPG, creatinine, UA and WBC as covariates together, increasing Lp(a) level was still positively correlated to CAD progression (OR 1.451, 95% CI 1.177–1.789; *p*<.001).

The association between baseline Lp(a) and CAD progression was also explored by categorizing the Lp(a) with a cutoff value of 300 mg/L. In unadjusted multivariable logistic regression analysis revealed that the odds ratio for CAD progression increased in patients with higher Lp(a) group (OR 1.530, 95% CI 1.075–2.177; *p* = .018).This positive correlation remained statistically significant after adjusting for age, sex, BMI, history of HTN, DM, initially obstructive CAD, smoking status, HbA1c, FPG, non‐HDL, HDL‐C, LDL‐C, creatinine, UA, and WBC (OR 1.642, 95% CI 1.018–2.649; *p* = .042). Results of logistic regression analyses are summarized in Table 3.

**TABLE 3:** Multivariate logistic regression analysis for CAD progression

	OR	95% CI	*p*
**Unadjusted Model**			
Continuous Lp(a)	1.287	1.101–1.503	.002^a^
Lp(a) >300 mg/L	1.530	1.075–2.177	.018^a^
**Adjusted Model 1**			
Continuous Lp(a)	1.284	1.095–1.506	.002^a^
Lp(a) >300 mg/L	1.530	1.066–2.195	.021^a^
**Adjusted Model 2**			
Continuous Lp(a)	1.330	1.120–1.580	.001^a^
Lp(a) >300 mg/L	1.654	1.112–2.461	.013^a^
**Adjusted Model 3**			
Continuous Lp(a)	1.451	1.177–1.789	<.001^a^
Lp(a) >300 mg/L	1.642	1.018–2.649	.042^a^

*Note*: Results of continuous Lp(a) were represented by OR per log‐unit increase. Model 1 was adjusted for age and sex. Model 2, further adjusted for BMI, history of HTN, DM, O‐CAD, and smoking status. Model 3, further adjusted for HbA1c, FPG, non‐HDL, HDL‐C, LDL‐C, Creatinine, UA, and WBC.

Abbreviations: BMI, body mass index; CI, confidence interval; DM, diabetes mellitus; FPG, fasting plasma glucose; HDL‐C, high‐density lipoprotein cholesterol; HTN, hypertension; LDL‐C, low‐density lipoprotein cholesterol; O‐CAD, initially obstructive coronary artery disease; OR, odds ratio; UA, uric acid; WBC, white cell counts.

^a^ *p*<.05.

### 3.5 Subgroup analysis for the relation of Lp(a) with CAD progression

**FIGURE 2:**
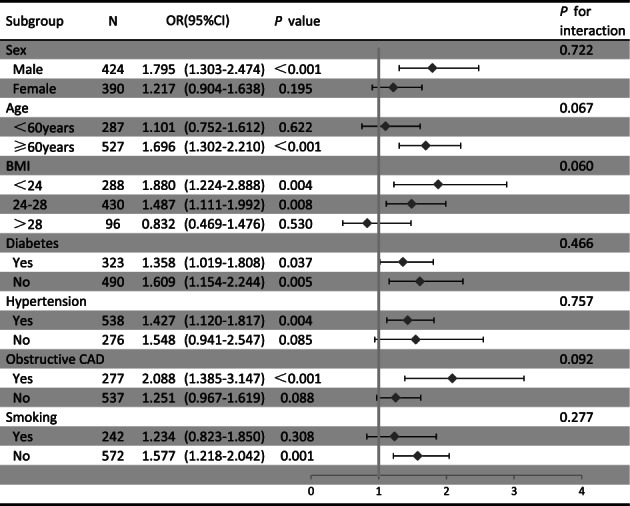
Subgroup analysis for the impact of log‐transformed continuous Lp(a) on CAD progression. OR, odds ratio; CI, confidence interval; BMI, body mass index; CAD, coronary artery disease

Further assessment of the association between baseline serum Lp(a) and CAD progression was performed in various subgroups of the study population(Figure 2). After adjustment of several covariates in Model 3, subgroup analysis indicated that subjects with aged ≥60 years, with lower BMI, with male gender, with initially obstructive CAD had higher odds ratios than those without these risk factors. Meanwhile, subjects with DM, with HTN, and with smoking status had lower odds ratios. However, the association of baseline Lp(a) and CAD progression seemed to be more prominent in subjects with lower BMI (OR [95% CI] BMI<24 kg/m^2^ 1.880 [1.224–2.888] vs. 24 kg/m^2^ ≤ BMI<28 kg/m^2^ 1.487 [1.111–1.992] and BMI≥28 kg/m^2^ 0.832 [0.469–1.476], *p* for interaction = 0.060).

## 4 DISCUSSION

In the present study, our results demonstrated that elevated baseline serum Lp(a) was positively associated with angiographic progression of coronary artery disease, particularly in patients with relatively lower BMI, independent of traditional cardiovascular risk factors, including age, sex, BMI, history of HTN, DM, initially obstructive CAD, smoking status, HbA1c, FPG, non‐HDL, HDL‐C, LDL‐C, creatinine, UA and WBC. Our findings contribute to a growing body of literature supporting the hypothesis that Lp(a) is correlated with progression of CAD.

Many previous studies have described the correlation between Lp(a) and coronary atherosclerosis progression using invasive imaging methods. In one study performed in 76 patients with acute coronary syndrome (ACS) who underwent intravascular ultrasound(IVUS) at baseline and at 10‐month follow‐up after percutaneous coronary intervention, the results showed that patients with serum levels of Lp(a) >20 mg/dl had slight plaque progression despite of statin therapy, suggesting Lp(a) levels could be an alternative predictor of further plaque progression.^10^ Similarly, Hartmann et al. reported that Lp(a) was positively related to plaque progression in the left main coronary artery by serial IVUS examinations.^18^ Chetan et al. also demonstrated that Lp(a) was positively and independently correlated with increased percent atheroma volume by using IVUS examination.^19^ Furthermore, a meta‐analysis with nine cohort studies including 1834 patients was conducted to evaluate the association between the baseline Lp(a) levels and the restenosis after successful coronary stenting. The results indicated that elevated baseline Lp(a) levels were positively associated with increased risk of in‐stent restenosis after successful coronary stent implantation.^20^ Moreover, Maya et al. found CAD patients with elevated LP(a) levels (>50 mg/dl) undergoing specific Lp(a) apheresis for 18 months produced regression of coronary atherosclerosis on the background of optimal medical therapy, suggesting Lp(a) could be a novel predictive and therapeutic biomarker for coronary atherosclerosis burden.^21^ In the present study, we analyzed the association between baseline serum levels of Lp(a) and progression of CAD by non‐invasive coronary CTA, which was considered as convenient tool and widely used in clinic. The Gensini score system was utilized to evaluate the severity of coronary lesions. According to the definition of progression described previously by Riyaz et al.,^17^ we revealed that angiographic progression of CAD positively associated not only with continuous serum levels of Lp(a) but also with Lp(a)>300 mg/L before and after adjustment of conventional risk factors, which was consistent with previous studies.

Conversely, Puri et al. found no evidence of a correlation of Lp(a) with coronary atheroma progression rate during 2 years high‐intensity statin therapy in sub‐analysis of the Study of Coronary Atheroma by Intravascular Ultrasound: Effect of Rosuvastatin Versus Atorvastatin (SATURN) trial.^9^ Their findings showed that neither baseline nor on‐treatment Lp(a) levels in patients with CAD under high‐intensive statin treatment had effect on changes in coronary atheroma volume. One possible explanation for the discrepancy between our study and the SATURN trial was racial difference, as 96% were Caucasian in SATURN trial. Previous study included 6086 patients with first myocardial infarction were stratified by race and adjusted for age and sex. The study found that increased Lp(a) levels were significantly associated with increased MI risk, particularly in South Asian and Latin American populations.^22^ Until now, racial difference in the correlation between Lp(a) and increased ASCVD risk remains controversial.^23^, ^24^ Meanwhile, differences in the enrolled patients could also account for the discrepancy. The present study group comprised patients with cardiovascular risk in both of primary and secondary prevention populations at outpatient department, while the SATURN trial screened subjects with stable CAD. Most of patients received moderate‐intensive statin therapy in our study, however, patients were given high‐intensive statin therapy in SATURN trial. The follow‐up LDL values were much lower in SATURN trial (rosuvastatin group 60.5 ± 25.9 mg/dl, atorvastatin group 67.2 ± 22.6 mg/dl), compared with those in our study (follow‐up total LDL‐C 105.2 ± 37.5 mg/dl). Our results showed that patients in “progressors” group had higher serum levels of Lp(a) than those in “non‐progressors” group despite of comparable follow‐up LDL‐C levels (105.5 ± 39.0 vs. 105.2 ± 36.7, *p* = 0.871), implying the potential predictive value of Lp(a) on the basis of comparable LDL‐C levels. Interestingly, a meta‐analysis with 11 studies including 18 979 patients was performed to assess the prognostic value of Lp(a), the authors confirmed that the correlation between Lp(a) and cardiovascular events may be attenuated in patients with lower levels of LDL‐C.^25^ Furthermore, it was found that the prognostic utility of Lp(a) as a marker of risk in the setting of primary and secondary prevention was apparently only in the population with higher cholesterol levels.^26^ However, a single‐center cross‐sectional study was recently conducted to investigate the association between Lp(a) and coronary atherosclerotic lesion using Gensini score, Li et al. found the association was influenced by LDL‐C levels. They revealed that Lp(a) was the risk factors of coronary atherosclerotic heart disease in patients with LDL‐C<100 mg/dl.^27^ Therefore, the evaluation of Lp(a) in patients with well controlled cholesterol remained to be further studied.

Another interesting finding of the present study was that the association of baseline Lp(a) and CAD progression seemed to be more prominent in subjects with relatively lower BMI, even after controlling for conventional covariates. In prior studies, obesity has been considered as an independent risk factor for CAD, as well as increased rates of adverse cardiovascular events.^28^ Current guidelines recommend weight control to be a fundamental life style changes strategy in management of CAD.^29^ Previously, Troy et al. conducted a prospective multicenter observational study to confirm that increased BMI had a strong and consistent relationship with the prevalence, extent, and severity of CAD.^30^ Afterward, Cho et al. analyzed the association between obesity type and CAD in stable symptomatic postmenopausal women. The authors revealed that central obesity but not overall obesity was related to obstructive CAD.^31^ As we know obesity usually accompanies with several clinical comorbidities, such as HTN, DM, and hyperlipidemia, which may categorize to the high risk individuals. Nowadays, many guidelines recommend to screen the Lp(a) level mostly in high‐risk individuals.^32^ However, the present study showed the association of baseline Lp(a) and CAD progression was more prominent in patients with relatively lower BMI rather than obesity, one possible explanation was that Lp(a) may play a significant role as an overlooked “residual risk factor,” which implying the importance of routine Lp(a) measurement to assist in early risk stratification in seemingly normal‐sized populations. Further studies are needed to confirm the relationship and reveal the underlying mechanism.

In the present study, there were several limitations. First, due to the variable numbers of the KIV type2 domains, method we used in the present study may be influenced by the apo(a) sizes. The results of Lp(a) levels may be overestimated or underestimated and may cause inaccurate evaluation of a patient's risk for CAD progression. Second, since this was a retrospective, longitudinal study conducted at a single‐center, residual confounders are hard to avoid. For example, diet, excise, alcohol consumption patterns, and genetic background were not monitored during the study, which was variable. Third, this study only enrolled patients who took repeated coronary CTA for suspected CAD, which could lead to a selection bias. Fourth, only quantitative CTA analysis and angiographic score were performed due to noninvasive and convenience for clinical application, qualitative analysis of coronary plaques was unavailable. Fifth, as Lp(a) was genetically determined by LPA gene, with little influence from lifestyle interventions, therefore, Lp(a) levels did not fluctuate significantly during the whole life.^33^ In the present study, we mainly aimed to analyze the correlation between baseline Lp(a) and coronary artery progression. In addition, 85% subjects received statins treatment in our study. Currently, statins effect on Lp(a) metabolism was still not well understood, resulting in controversial results by different statins.^5^, ^34^ Whether changes in Lp(a) levels over time could affect the result will need additional study. Thus, further studies are required in multicenter and larger populations to confirm our findings, especially therapeutic strategies of Antisense oligonucleotide (ASO) technology.

## 5 CONCLUSION

In conclusion, the current study found that baseline serum Lp(a) concentration was significantly associated with angiographic progression of CAD, particularly in those with relatively low BMI. Therefore, Lp(a) could be a potent risk factor for progression of CAD, assisting in early risk stratification in cardiovascular patients. Further studies will clearly be required to confirm our findings.

## CONFLICT OF INTEREST

No conflict of interest was declared.

## AUTHOR CONTRIBUTIONS

Conceived and designed the study: Lin Chen and Zhen Wu; clinical data acquisition: Binghan Zheng and Yongxia Wu; data analysis: Xujing Xie, Zefeng Chen and Zhen Wu; statistical analysis: Xing Shui and Zheqi Wen; Manuscript drafting: Xing Shui, Zheqi Wen and Zefeng Chen; Each author contributed important intellectual content during manuscript writing or revision.

## Supporting information


**Supplementary Table S1** Baseline clinical and biochemical data according to the CAD progression

## Data Availability

The data that support the findings of this study are available on request from the corresponding author. The data are not publicly available due to privacy or ethical restrictions.
