# Supplementary material for: Elevated serum lipoprotein(a) is significantly associated with angiographic progression of coronary artery disease
Source: Clin Cardiol. 2021 Aug 25;44(11):1551–9. doi: 10.1002/clc.23718 (PMC8571555; doi:10.1002/clc.23718)
Supplement: Supplementary file 1 — Supplementary Table S1 Baseline clinical and biochemical data according to the CAD progression [file CLC-44-1551-s001.docx]

Supplementary Table 1. Baseline clinical and biochemical data according to the CAD progression

| Variables | Progressors | Non-progressors | *P* |
| --- | --- | --- | --- |
|  | n=258 | n=556 |  |
| Age, year | 65.70±10.03 | 62.07±10.86 | ＜0.001^*^ |
| Male, n(%) | 152(58.9) | 272(48.9) | 0.008^*^ |
| BMI,kg/m2 | 24.29±2.97 | 24.61±3.08 | 0.171 |
| Smoking, n(%) | 86(37.2) | 127(26.1) | 0.002^*^ |
| HTN, n(%) | 188(72.9) | 350(62.9) | 0.005^*^ |
| DM, n(%) | 113(44.0) | 210(37.8) | 0.093 |
| O-CAD, n(%) | 106(41.1) | 171(30.8) | 0.004^*^ |
| **Laboratory tests** |  |  |  |
| TC, mmol/L | 4.95±1.30 | 4.93±1.11 | 0.841 |
| TG, mmol/L | 2.11±2.38 | 1.82±1.25 | 0.064 |
| HDL-C, mmol/L | 1.11±0.29 | 1.20±0.31 | ＜0.001^*^ |
| LDL-C, mmol/L | 3.05±1.01 | 3.07±0.94 | 0.799 |
| LDL-C,follow-up, mmol/L | 2.73±1.01 | 2.72±0.95 | 0.871 |
| Non-HDL, mmol/L | 3.84±1.26 | 3.73±1.03 | 0.244 |
| ApoA1, g/L | 1.32±0.28 | 1.36±0.26 | 0.041^*^ |
| ApoB100, g/L | 1.10±0.47 | 1.03±0.32 | 0.034^*^ |
| Lp(a), mg/L | 161.50(81.25,301.40) | 121.80(64.55,231.23) | 0.001^*^ |
| Lp＞300mg/L, n(%) | 66(25.6) | 102(18.3) | 0.018^*^ |
| FPG, mmol/L | 6.45±3.97 | 6.18±2.30 | 0.236 |
| HbA1c, (%) | 6.91±1.90 | 6.84±1.50 | 0.005^*^ |
| Creatinine, umol/L | 77.64±20.94 | 75.21±19.60 | 0.110 |
| eGFR, mL/min/1.73m2 | 87.06±45.92 | 83.83±47.45 | 0.361 |
| UA, umol/L | 380.30±116.03 | 366.61±109.10 | 0.106 |
| WBC, ×10E9/L | 7.03±2.24 | 6.79±1.41 | 0.066 |
| **Medication** |  |  |  |
| Anti-platelet drug, n(%) | 229(88.8) | 466(83.8) | 0.063 |
| Statins, n(%) | 223(86.4) | 469(84.4) | 0.439 |
| ACEI/ARB, n(%) | 179(69.4) | 355(63.8) | 0.122 |
| β-blocker, n(%) | 155(60.1) | 299(53.8) | 0.092 |
| OAD, n(%) | 92(35.7) | 177(31.8) | 0.280 |
| Insulin, n(%) | 48(18.6) | 76(13.7) | 0.068 |

BMI, body mass index; O-CAD, initially obstructive coronary artery disease; SBP, systolic blood pressure; DBP, diastolic blood pressure; HR, heart rate; TC, total cholesterol; TG, triglycerides; HDL-C, high-density lipoprotein cholesterol; LDL-C, low-density lipoprotein cholesterol; FPG, fasting plasma glucose; eGFR, estimated glomerular filtration rate; UA, uric acid; WBC, white cell counts; AECI, angiotensin converting enzyme inhibitors; ARB, angiotensin receptor blocker; OAD, oral anti-diabetic drugs.

^*^P＜0.05.
